# Coadministration of Nematophagous Fungi for Biological Control over Nematodes in Bovine in the South-Eastern Brazil

**DOI:** 10.1155/2018/2934674

**Published:** 2018-03-26

**Authors:** Fábio Dias Luns, Rafaela Carolina Lopes Assis, Laryssa Pinheiro Costa Silva, Carolina Magri Ferraz, Fábio Ribeiro Braga, Jackson Victor de Araújo

**Affiliations:** ^1^Department of Veterinary Medicine, Laboratory of Parasitology, Federal University of Viçosa, Av. P.H. Rolphs, Viçosa Campus, 36570-000 Viçosa, MG, Brazil; ^2^Department of Veterinary Medicine, University Vila Velha, Avenue Comissário José Dantas de Melo, 21, Boa Vista, 29102-920 Vila Velha, ES, Brazil

## Abstract

This study compared the coadministration among the three nematode predatory fungi,* Duddingtonia flagrans*,* Monacrosporium thaumasium*, and* Arthrobotrys robusta*, in the biological control of cattle gastrointestinal nematodiasis in comparison with the use of the fungus* D. flagrans* alone. Five groups consisting of eight Girolando heifers were kept in paddocks of* Brachiaria decumbens* for six months. Each heifer received 1 g/10 kg of pellets containing the fungi (0.2 g of fungus/10 kg b.w.). Group 1 (G1) received pellets with* D. flagrans* and* M. thaumasium* in coadministration, G2 received* D. flagrans* and* A. robusta*, G3 received* M. thaumasium*,* A. robusta*, and* D. flagrans*, and G4 received the fungus* D. flagrans* alone. Group 5 (control) received pellets without fungi. The monthly mean of fecal egg count (FEC) of Groups 1, 2, 3, and 4 were 93.8, 85.3, 82.7, and 96.4% smaller than the mean of control group. The treatments with pellets containing* D. flagrans* or* D. flagrans* +* M. thaumasium* produced significantly better results than the* D. flagrans* +* A. robusta* or the combination of the three fungi. The associations which include* A. robusta* were less efficient in this study than* D. flagrans* alone or associated with* M. thaumasium.*

## 1. Introduction

Nematode-trapping fungi are the most studied group of fungal nematode antagonists. They have the greatest potential for destroying infectious forms of gastrointestinal nematode parasites among both animals and humans [1, 2]. The fungal species* Duddingtonia flagrans*,* Monacrosporium thaumasium*, and* Arthrobotrys robusta* were identified as predators of nematodes and they have been studied as biological control agents for these parasites [3–5]. Research on the application of the nematode-trapping fungi* D. flagrans* [6, 7],* A. robusta* [5, 8], and* M. thaumasium* [9, 10] in the treatment of gastrointestinal nematodiasis in bovines has demonstrated the potential of these fungi in reducing the free-living stages of parasitic nematodes (L3) in the field. The use of more than one biocontrol agent is considered a primary suppressive measure that contributes to controlling the presence of infectious agents in soil [11]. The use of a combination of several nematophagous fungi can minimize any potential flaws in their individual administration, or it may even enhance their actions as biocontrol agents [12]. Furthermore, numerous biological control mechanisms (including the production and use of substances that exert fungicidal effects) may vary among species and even among isolates of the same species, resulting in interactions between fungi that may interfere with their antagonistic performance [13]. However, most of the studies examining biological control in cattle have been conducted with fungal isolates applied alone. There are no reports of previous* in vivo* studies that have evaluated the biological control achieved following coadministration of* D. flagrans, A. robusta*, and* M. thaumasium* in extensive systems of dairy cattle. It is unclear whether the coadministration of a few of these species could yield additive effects from a biological standpoint [14]. Many studies have already proven the effectiveness of the fungus.* D. flagrans* in the biological control of nematodes of several species [1–3, 6, 7]. We then chose this fungus to compare the treatment groups with fungal association. In this context, the objective of this study was to evaluate the effects of the coadministration of nematode-trapping fungi* A. robusta, D. flagrans*, and* M. thaumasium* on gastrointestinal helminths in combination would be synergistic or antagonistic in nature compared to* Duddingtonia flagrans* used alone.

## 2. Materials and Methods

### 2.1. Area of Study

The experiment was carried out at a private farm located in the municipality of Ouro Branco, state of Minas Gerais, in the south-eastern region of Brazil, 20°31′15′′ south latitude and 43°41′31′′ west longitude, from April to September 2012. The paddock's topography is undulating to hilly (5% flat, 60% undulating, and 35% hilly), with an mean altitude of 1,052 m (maximum: 1,568 m; minimum: 1,099 m) and featuring native vegetation indicative of a transition zone between the Atlantic forest and savanna. The climate is tropical (Köppen-Geiger climate classification: Aw), with an annual mean maximum temperature of 22°C and a minimum temperature of 7°C and featuring an mean annual rainfall of 1,200 mm.

### 2.2. Fungi and the Production of a Mycelial Mass

Isolates of three fungal species that are known predators of nematodes were used:* A. robusta* (I31),* D. flagrans* (AC001), and* M. thaumasium* (NF34). These isolates were obtained from soils in the Zona da Mata region of the state of Minas Gerais, Brazil. Mycelium was obtained by transferring disks (~4 mm in diameter), which were cultured with the fungal isolates in 2% water agar (2% WA), to Erlenmeyer flasks (250 mL in capacity) containing 150 mL of liquid glucose-yeast-peptone (GYP) medium [3]. These flasks were then incubated in the dark, under agitation at 120 rpm, at 26°C for 10 days. Following this period, the fungal mycelium was removed and weighed on an analytical balance. All of the procedures followed the methodology of Araújo et al. [10].

### 2.3. Experimental Animals

At the beginning of the experiment, a total of 40 6-month-old Girolando heifers, with an mean body weight (b.w.) of 120 kg, were pretreated with 10% albendazole (Mogivet Lab®, Brazil), which was orally administered at a dose of 1 mL/20 kg of b.w. Fifteen days after the antihelminthic treatment, the heifers were separated into one of five groups (Groups 1, 2, 3, 4, and 5) consisting of eight heifers each, based on the animals' mean weight.

The heifers were allocated in five paddocks of* Brachiaria decumbens* that had been previously grazed by young and adult animals and which were naturally infected with gastrointestinal helminth parasites. Each paddock had an area of 15 ha. Each group was allocated in only one paddock without rotational grazing during the experimental period. Each animal from all groups treated (G1, G2, G3, and G4) received 1 g of pellets (0.2 g of fungal mycelium) per 10 kg of b.w. The animals from Group 5 received 1 g of fungus-free pellets per 10 kg of b.w. All of the animals received the pellets orally twice a week. The pellets were mixed in a concentrated and balanced ration provided for dairy cattle (accounting for 18% of the cattle's total protein, Federal University of Viçosa). The cattle were given water ad libitum for 6 months, starting from April 2012.

The differences between the groups were in the composition of the pellets. Pellets of group 1 (G1) contained the fungi* D. flagrans* and* M. thaumasium*, while the pellets of Group 2 (G2) contained the fungi* D. flagrans* and* A. robusta*. Group 3 (G3) received pellets containing the three fungi* D. flagrans*,* A. robusta*, and* M. thaumasium*. Group 4 (G4) pellets contained only the* D. flagrans* fungus. The pellet doses of the different groups were all comparable; with respect to the proportions of fungi species included in each pellet, Groups 1 and 2 contained 50% of each of two fungi, while the pellets combining the three fungal isolates (Group 3) were comprised of one-third of each fungus.

After allocating the heifers to the paddocks, the animals' fecal samples were collected directly from the rectum, once a week, to determine the number of nematode fecal egg count (FEC), as described by Gordon and Whitlock [15].

Meteorological data were recorded daily at a specialized station in the region; the mean of the maximum, mean, and minimum monthly temperatures, as well as the mean rainfall, were noted.

Fecal samples were collected to observe fungal growth once a week, 2 days after the animals were treated with the fungi. The feces were incubated in plates containing 2% WA; 100 L3 were recovered from the coproculture and they were placed into a drying oven at 25°C for 10 days to confirm the passage and predatory ability of the fungi through the cattle's gastrointestinal tract, as well as to assess fungal growth in the feces [3].

Coproculture was evaluated together with FEC counts; 20 g of feces was mixed with autoclaved wood shavings and kept moist at a controlled temperature (25°C) for 7 days to obtain trichostrongylid larvae. Identification of the infective larvae in the coproculture was performed according to Keith [16].

The FEC and larvae recovered from the coproculture of animals in both the treated and control groups were recorded, and the percentage of larval reduction was determined according to De Gives et al. [17]: reduction (%) = mean L3 recovered from control group − mean L3 recovered from treated group × 100/mean L3 recovered from the control group.

The FEC, number of infective larvae recovered from the feces, were statistically analyzed on a weekly basis and compared over the experimental period.

The data were transformed into log⁡(*x* + 1) prior to the analysis and subjected to analysis of variance (ANOVA) with repeated measures, Tukey's test, and regressions using a randomized design at probability levels of 1% and 5%.

The animals' weights were also compared throughout the experiment, starting from April 2012. The correlation analyses were performed using Pearson's correlation (*P* < 0.001). The analyses were performed using the BioEstat 3.0 Software.

The Ethics Committee of the Federal University of Viçosa protocol number 66/2012 approved this study.

## 3. Results

**Figure 1:**
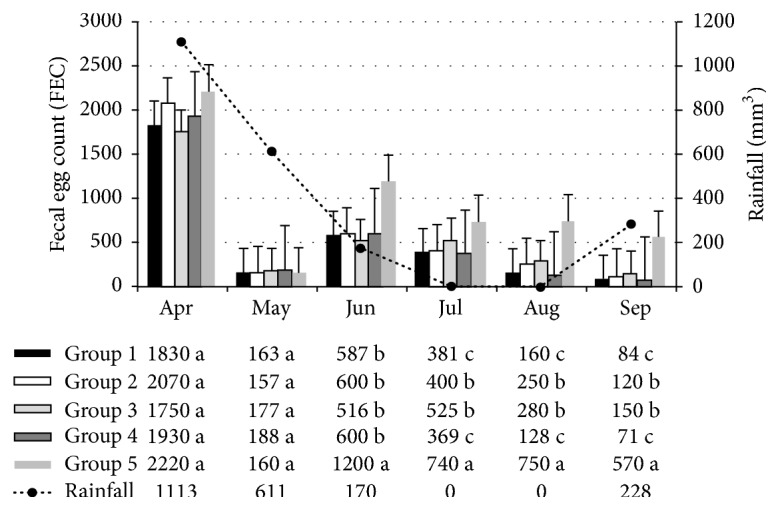
Monthly mean of the fecal eggs count (FEC) among heifers in the groups treated with various combinations of coadministered nematophagous fungi:* D. flagrans* +* M. thaumasium* (G1);* D. flagrans* +* A. robusta* (G2);* D. flagrans* +* M. thaumasium* +* A. robusta* (G3);* D. flagrans* alone (G4); and the control group. All samples were collected from April to September 2012 in Ouro Branco, MG, Brazil. (^a, b, c^Numbers followed by different letters present statistical difference.)

The monthly mean values of FEC counts are shown in Figure 1. In the first month of the experiment, no statistically significant differences were found (*P* > 0.05) between the groups treated with fungi (G1, G2, G3, and G4) and the control group (group 5). In the first month of treatment (May 2012), the low FEC number was likely due to the previously administered anthelmintic treatment. The FEC of animals treated with* D. flagrans *and* M. thaumasium* (Group 1);* D. flagrans* and* A. robusta* (Group 2);* D. flagrans*,* A. robusta*, and* M. thaumasium* (Group 3); and* D. flagrans* (Group 4), that is, all treated groups, were significantly lower than those of the control group from June to September 2012 (*P* < 0.05). However, the FEC of the animals treated with* D. flagrans* and* M. thaumasium* (Group 1) and with* D. flagrans* alone (Group 4) were significantly lower than those treated with* D. flagrans *and* A. robusta* (Group 2) and with* D. flagrans*,* A. robusta*, and* M. thaumasium* (Group 3) (*P* < 0.05) (Figure 1).

The monthly mean FEC of the animals in the group treated with pellets containing the fungus* D. flagrans* alone, as well as those treated with pellets containing both* D. flagrans* and* M. thaumasium*, were 96.4% and 93.8% lower, respectively, than the FEC of the animals in the control group at the end of the experiment. The animals from Group 2, which were treated with pellets containing the fungi* D. flagrans* and* A. robusta*, as well as the animals from Group 3 (treated with pellets containing the three fungi), exhibited FEC reductions of 85.3% and 82.7%, respectively, when compared with the animals in the control group. Moreover, the FEC counts were significantly lower in Groups 1 and 4 when compared with Groups 2 and 3 at the end of the experiment (*P* < 0.05).

**Figure 2:**
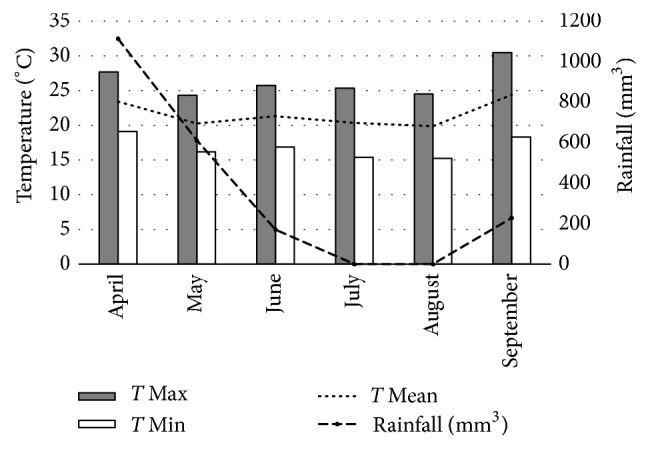
The mean of the maximum, mean, and minimum monthly temperatures (°C) and monthly rainfalls (mm^3^) recorded from April to September 2012, Ouro Branco, MG, Brazil.

Figure 2 shows the maximum, mean, and minimum temperatures, as well as the mean monthly rainfall. Overall, it was found that the meteorological data correlated with the parasitological findings, as temperatures and rainfall influenced the environmental parasite load.

The coproculture showed that the* Cooperia* sp. was the most prevalent gastrointestinal parasitic nematode in all groups throughout the experiment, which was observed at percentages of 68%, 67.6%, 60.6%, 46.2%, and 45.1% for Groups 1, 2, 3, 4, and 5, respectively; this was followed by* Haemonchus*, which was found at rates of 22.8%, 24.8%, 30.4%, 45.5%, and 45.8%, and* Oesophagostomum*, which was observed at rates of 9.2%, 8.4%, 8.4%, 9.2%, and 8.7%, respectively. No significant differences (*P* > 0.01) were found with respect to the proportion of the different genera among the five groups. The percent reduction of L3 recovered from each coproculture of the treated groups was significantly lower when compared with that of the control group. The reductions were 92.3%, 90.7%, 81.5%, and 78.3% for Groups 1, 2, 3, and 4, respectively.

Analysis of the culture plates confirmed the fungal growth, the specific conidia of each isolate, and the ability of* D. flagrans*,* A. robusta*, and* M. thaumasium* to predate L3 in all treated groups, confirming the passage of the isolates through the animals' gastrointestinal tracts. The presence of nematophagous fungi was not detected in the feces of the control group animals during the experiment.

**Figure 3:**
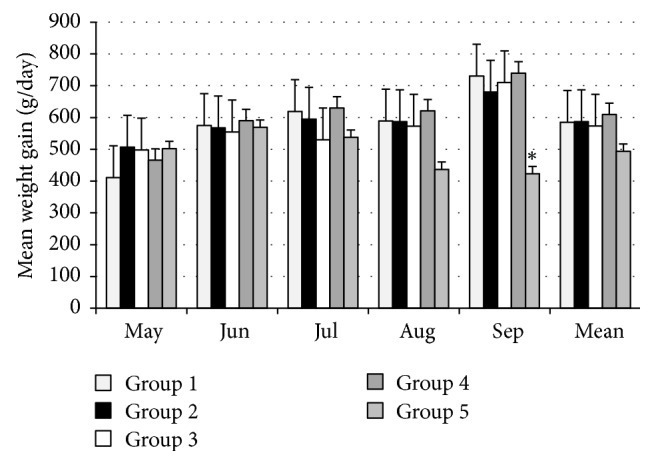
Mean weight gains (g/day) from each group. Measurements were taken from April to September 2012 in Ouro Branco, MG, Brazil. Significant differences between each treated group and the control group are indicated by an asterisk (Tukey's test).

Figure 3 shows the mean weight gains for the animals in the five groups. The weight gains of the animals in the treated groups (G1, G2, G3, and G4) differed from those of the animals in the control group (G5) (*P* < 0.05) in the last month of the study.

## 4. Discussion 

Studies evaluating the coadministration of nematophagous fungi are scarce, and this work was the first to evaluate the combined use of the fungi* A. robusta*,* D. flagrans*, and* M. thaumasium* in bovines. In this study, the heifers from the group treated with* D. flagrans* alone exhibited an FEC reduction of 96.4% when compared with the heifers in the control group. Several studies using the fungus* D. flagrans* in ruminants also reported smaller monthly mean FEC counts among the treated animals in relation to the control group [3, 18–20]. In studies using the same* D. flagrans* isolate, in Brazil, others researchers also obtained significant reductions in FEC in treated crossbred Holstein-Zebu and Nellore bulls, 31% and 57%.

There was a 93.8% reduction in the FEC among the group treated with* D. flagrans* and* M. thaumasium* when compared with the FEC of the control group. Studies using* M. thaumasium* in bovines reported that the monthly mean FEC were lower in treated animals. In studies where crossbred Holstein-Zebu heifers and Nellore bulls were treated with this same* Monacrosporium thaumasium* isolate, the authors obtained FEC reductions of 88.8% and 47.8%, respectively [3, 9].

Furthermore, the coadministration of pellets containing* D. flagrans* and* M. thaumasium* employing the same formulation as that used in this experiment was tested in sheep and the author demonstrated that this treatment was effective in controlling gastrointestinal helminths in young and adult sheep in the semiarid region of northeastern Brazil [21]. In addition, the researchers found that the FEC rates remained statistically significantly lower throughout the study without the administration of salvage deworming, reaching a 76% reduction in the FEC of treated animals when compared with the FEC of controls, which still required to be dewormed seven times [21].

In this study, the group treated with* D. flagrans* and* A. robusta* showed an FEC reduction of 85.3% in relation to that of the control group. Other study reported a 51.9% reduction in the FEC of crossbred Holstein-Zebu calves treated with an isolate of* A. robusta*.

Moreover, the compatibility between isolates* A. robusta* and* D. flagrans* was evaluated under laboratory conditions. With the aid of direct confrontation and antibiosis and volatile metabolite tests, the authors verified that the* A. robusta* isolate colonized approximately two-thirds of the plate, suggesting that there was competition (and subsequent antagonism) between these two fungi. Specifically,* A. robusta* reduced the growth of* D. flagrans*, suggesting the action of volatile antibiotics in inhibiting mycelial growth. These results corroborate the findings of the* in vivo* experiment described herein, since the groups of animals that received the coadministration treatments containing the* A. robusta* isolate demonstrated lower nematode reduction results when compared to the other treatments with statistical significance (*P* < 0.05).

The heifers in the group treated with the combination of the three fungi had an FEC reduction of 82.7% when compared with the animals in the control group. There are no previous records in the literature describing the coadministration of the three nematophagous fungi in the biological control of nematode parasites* in vivo*. This is the first report of its kind; the fungal combinations that were tested were effective in reducing the FEC in cattle. Furthermore, the coadministration of these three isolates was less effective than the coadministration of* D. flagrans* and* M. thaumasium*, or the administration of the* D. flagrans* isolate alone, at reducing the FEC.

Trap formation and L3 predation by fungal isolates were confirmed by* in vitro* assays. Braga et al. [1] reported that* D. flagrans* showed greater predatory activity* in vitro* (80.3%) on L1* Angiostrongylus vasorum* when compared with* M. thaumasium* (74.5%) and* A. robusta* (71.8%). In another* in vitro* study, Braga et al. [22] compared the predatory ability of the same isolates used in this study on L3* Strongyloides stercoralis*. The L3 reductions were 83.7%* (D. flagrans)*, 75.5%* (M. thaumasium)*, and 73.2%* (A. robusta)*. A study conducted to examine the interaction between L3* H. contortus* in goat and the fungi* M. thaumasium* and* A. conoides* showed that both strains were able to reduce the larval population, but* M. thaumasium* proved to be more efficient [23]. These results are in agreement with the findings of this work; the greatest reduction in L3 observed at the end of the experiment was due to* D. flagrans*, while combinations featuring* A. robusta* were less efficient at reducing L3 and the FEC counts.

In the present study, the percent reduction of L3 achieved by the* M. thaumasium* isolate alone was 92.3%. Similarly, Araújo et al. [10] recorded larval reduction in the coproculture of animals treated with* M. thaumasium* in the Brazilian semiarid region. In their study, the combination of* D. flagrans* and* M. thaumasium* resulted in a 90.7% decrease in the number of larvae in the coproculture.

Furthermore, the* in vitro* action of fungal isolates* A. robusta* and* M. thaumasium* on L3 cyathostome in horses was compared and the percent reduction obtained with* M. thaumasium *313 (93.4%) was higher than that obtained with the* A. robusta *isolate (86.3%) at 25°C. The combination of the same isolates (*A. robusta* +* M. thaumasium*) in this study reduced the L3 recovered in the coproculture by 81.5%, while a combination of isolates* A. robusta* +* M. thaumasium* + the fungus* D. flagrans* decreased L3 by 78.3%.

It is important to note that climate plays an important role in the ability of fungi to trap nematodes, particularly since the optimum growth temperature varies with each fungal species. Morgan et al. observed that temperatures in the range of 20°C–33°C influenced the larvae trapping percentage in different species of fungi [24]. Thus, optimal rainfalls and predation temperatures, that is, specific environmental climatic conditions, may be directly related to the results of this study. To support this, Castro et al. reported that* A. robusta* exhibited the best larval trapping results at temperatures ranging from 25°C to 28°C, while* M. thaumasium* was not affected by temperatures of 25°C–30°C, which confirms that temperature influences the degree of trapping, depending on the cyathostome species or genus [25].

In addition, Castro et al. obtained a 93.36% reduction in the number of cyathostome larvae after administering* M. thaumasium* at 25°C. The authors found that temperatures of 25°C, 28°C, and 30°C did not affect the performance of* M. thaumasium*, which demonstrated an mean efficiency of 94% [25]. The results obtained in our study corroborate the findings of these previous works and suggest that the efficiency with which larvae are controlled by nematophagous fungi essentially depends on the choice of the fungal species, as well as on their suitability for specific temperature conditions.

The fungus* Monacrosporium* demonstrated unvarying performance* in vitro* at a temperature range of 15°C–30°C, as reported by Mendoza-de Gives and Vázquez-Prats [26] and Castro et al. [25] even at 30°C, a common temperature in the tropics; thus this fungi would be better adapted to the conditions of the Brazilian climate. These results may support and explain the higher degree of efficiency exhibited by the combination* M. thaumasium* and* D. flagrans* in reducing the FEC and L3 in this study, particularly when compared with the administration of* A. robusta* and* D. flagrans*. Moreover, the results also corroborate the reduced percentage of L3 observed in the coproculture after administering combinations featuring* A. robusta*. In this study, the region's temperatures ranged from 24.3°C to 30.5°C, which may be considered unsuitable for optimal predation by* A. robusta*.

Of note, all of the treated groups showed a similar pattern of weight gain during the study. Even though notable weight gain differences were found between heifers in the treated groups, there was a significant difference (*P* < 0.01) in weight gain between the treated groups, which varied in an inversely proportional manner to both the number of larvae recovered in pasture and the FEC. Specifically, the heifers treated with* D. flagrans* and* D. flagrans + M. thaumasium* showed greater mean weights. This reinforces the fact that administering pellets containing fungi was favored when the animals were pretreated. Moreover, greater weight gains in the treated animals (as compared to those in the control group) were also observed by Araújo et al. when testing the fungus* M. thaumasium* in goats in the Brazilian semiarid region. Furthermore, Braga et al. [1] studied horses in the field; the researchers administered the nematophagous fungus* D. flagrans*, and they observed significant weight gain differences between the groups treated with the fungus and the control animals. The animals that were treated with the fungus demonstrated greater weight gains than those in the control group.

## 5. Conclusion

Treating dairy cattle with alginate pellets containing, and coadministering, the nematophagous fungi* D. flagrans*,* A. robusta*, and* M. thaumasium* resulted in the biological control of gastrointestinal nematodes in bovines, although the level of control was not increased when compared with the use of the fungus* D. flagrans* alone. Coadministration with the* A. robusta* isolate was not considered a good alternative. The administration of* D. flagrans* alone was found to be more promising than coadministration for continuous use in dairy cattle in this tropical region in Brazil.
